# An Overview of Epidemiological Tools That Support the Progress of Foot-and-Mouth Disease Control in Southeast Asia

**DOI:** 10.3390/pathogens15090893

**Published:** 2026-08-25

**Authors:** Umanga Gunasekera, Nguyen Thi Diep, Pham Thanh Long, Vo Dinh Chuong, Jonathan Arzt, Andres Perez

**Affiliations:** 1 Department of Veterinary Population Medicine, College of Veterinary Medicine, University of Minnesota, Saint Paul, MN 55108, USA; vo000132@umn.edu (V.D.C.); aperez@umn.edu (A.P.); 2 Department of Animal Health and Production, Ministry of Agriculture and Environment, Hanoi 10000, Vietnam; 3 Foreign Animal Disease Research Unit, Agricultural Research Service, United States Department of Agriculture, Plum Island Animal Disease Center, National Bio and Agro-Defense Facility, Orient Point, NY 11957, USA

**Keywords:** foot-and-mouth disease, progressive control pathway, epidemiological evidence

## Abstract

Foot-and-mouth disease (FMD) causes significant losses for livestock farmers in affected countries and regions, including Southeast Asia, where several countries are progressing through different stages of the Progressive Control Pathway (PCP) under diverse resource settings. The PCP, developed by the World Organization for Animal Health and the Food and Agriculture Organization of the United Nations, is an outcome-oriented strategy designed to support progress toward the elimination of FMD. In this review paper, we propose an evidence-based framework linking epidemiological tools to each PCP stage requirements based on studies conducted in eight Southeast Asian countries through peer-reviewed research and gray literature. The findings indicate that Stage 1 progression is supported through risk-factor analyses, seroprevalence studies, and participatory epidemiology, while Stage 2 relies on spatial analyses and evaluations of control strategies. Progression to Stage 3 requires evidence of active surveillance systems for rapid detection of new viral incursions, whereas later stages depend on risk assessment, sustained surveillance, and restricting transboundary animal movement. Regional challenges include underreporting, surveillance-system deficiencies, and inconsistent vaccination monitoring. The framework proposed herein provides a practical approach for aligning the generation of evidence with PCP requirements. Ultimately, strengthened regional collaboration that coordinates transboundary risk management is crucial for progress toward sustainable FMD control in Southeast Asia.

## 1. Introduction

Foot-and-mouth disease (FMD) is a contagious disease that affects cattle, buffalo, and many other ruminants and suids. Although FMD infection typically results in low mortality, the disease causes considerable economic losses to farmers due to a combination of direct and indirect costs [1]. The Food and Agriculture Organization (FAO) of the United Nations and the World Organization for Animal Health (WOAH) have introduced an outcome-oriented framework for FMD control referred to as the Progressive Control Pathway (PCP). The PCP acknowledges that approaches vary from country to country to achieve key outcomes at different stages.

Countries enter PCP Stage 1 after completing an FMD risk assessment. The primary objective of this stage is to establish the foundations for disease control. Progression to Stage 2 requires the development of a strategic control plan with monitoring and evaluation systems. In Stage 3, countries establish an official FMD control program and thereby strengthen surveillance and risk-based interventions. Successful completion of Stage 3 enables countries to seek WOAH endorsement and advance to Stage 4. The PCP outlines several key points that should be fulfilled at each stage and relies on a self-evaluation process before being accredited by WOAH to obtain the official status for a given stage [2]. The PCP pathway relies on self-evaluation, and countries may lack evidence to demonstrate their progress.

Several reviews on FMD control in Southeast Asia (SEA) have identified the importance of the PCP pathway, underlying factors contributing to endemicity, and recommendations related to control programs focusing on vaccination while emphasizing the importance of regional support [3,4,5,6,7]. Yet these studies have not provided a specific framework to progressively control FMD across the region. In this review, we specifically focus on analytical tools to effectively demonstrate evidence of the progress made in each stage to move to the next stage.

We focused on Cambodia, Lao PDR, Myanmar, Malaysia, Thailand, the Philippines, Vietnam and Indonesia. The Philippines is presently free from FMD. Indonesia reported FMD in 2022 after being FMD-free for nearly 32 years and is now identified as being in Stage 2. Thailand is in Stage 4, with an officially endorsed FMD control program from the WOAH. Malaysia is in Stage 3, while both Myanmar and Laos are in Stage 2, developing a strategic risk-based FMD control plan, and Cambodia is in Stage 1 [8]. Vietnam is currently in Stage 3 and has requested official endorsement from the WOAH to advance to Stage 4 [9].

The overarching objective of this study is to propose an evidence-based framework for FMD control in SEA using specific epidemiological tools to reflect progress across different PCP stages.

## 2. Methods

This narrative review includes a structured literature search aimed at examining the epidemiological tools applied in SEA to generate evidence supporting activities through the various stages of the FMD Progressive Control Pathway (PCP) [10].

The primary inclusion criterion for sources of evidence was English-language studies that were published between 1 January 2010 and 30 July 2025. The SEA region includes the countries of ‘Vietnam’, ‘Cambodia’, ‘Lao PDR’, ‘Myanmar’, ‘Malaysia’, ‘Thailand’, ‘Philippines’, and ‘Indonesia’ for the outcome of ‘foot-and-mouth disease’. The MeSH terms (“Foot and Mouth Disease” OR FMD) AND (“Southeast Asia” OR Thailand OR Vietnam OR Cambodia OR Laos OR Myanmar OR Malaysia OR Indonesia) AND (PCP-FMD OR surveillance OR “risk factor” OR epidemiology OR modeling OR “seroprevalence” OR GIS OR “risk mapping”) were used to search the Web of Science.

Apart from the Web of Science, gray (non-peer reviewed) literature was searched for resources on the FAO website and the WOAH/FAO FMD Reference Laboratory Network to identify relevant studies for the same MeSH terms ‘Foot and Mouth Disease’ OR FMD and ‘South East Asia’, OR ‘SEA’. Terms “surveillance”, “risk factor”, “modeling”, “seroprevalence”, “GIS”, and “risk mapping” were also included. Regional FMD Roadmaps meetings, Global Coordination Committee on Foot and Mouth Disease (GCC-FMD) meeting reports, and FMD quarterly reports were identified as related to FMD reporting and surveillance. Regional FMD roadmap meetings on global FMD control strategy applied to SEA, such as the 27th SEACFMD National Coordinators Meeting reports and the global FMD control strategy reports, are widely referenced in this review. GCC-FMD reports were mainly focused on reviewing recommendations related to regional collaborations, public–private partnerships, and action plans generalized to all regions, such as Asia, the Americas, Africa, and West Eurasia, were not considered. FMD quarterly reports that track FMDV movement quarterly based on EMPRES-i, WOAH WAHID, and WRLFMD resources were not included in this review. FMD and similar transboundary animal disease events and information (FAST) reports, as well as standing technical-committee open-session meetings that provided technical and scientific information globally, were also not considered. Local administrative data were obtained from the Department of Animal Health and Production, Ministry of Agriculture and Environment, Hanoi, Vietnam, and included information related to the Vietnam FMD National Control Program.

**Figure 1:**
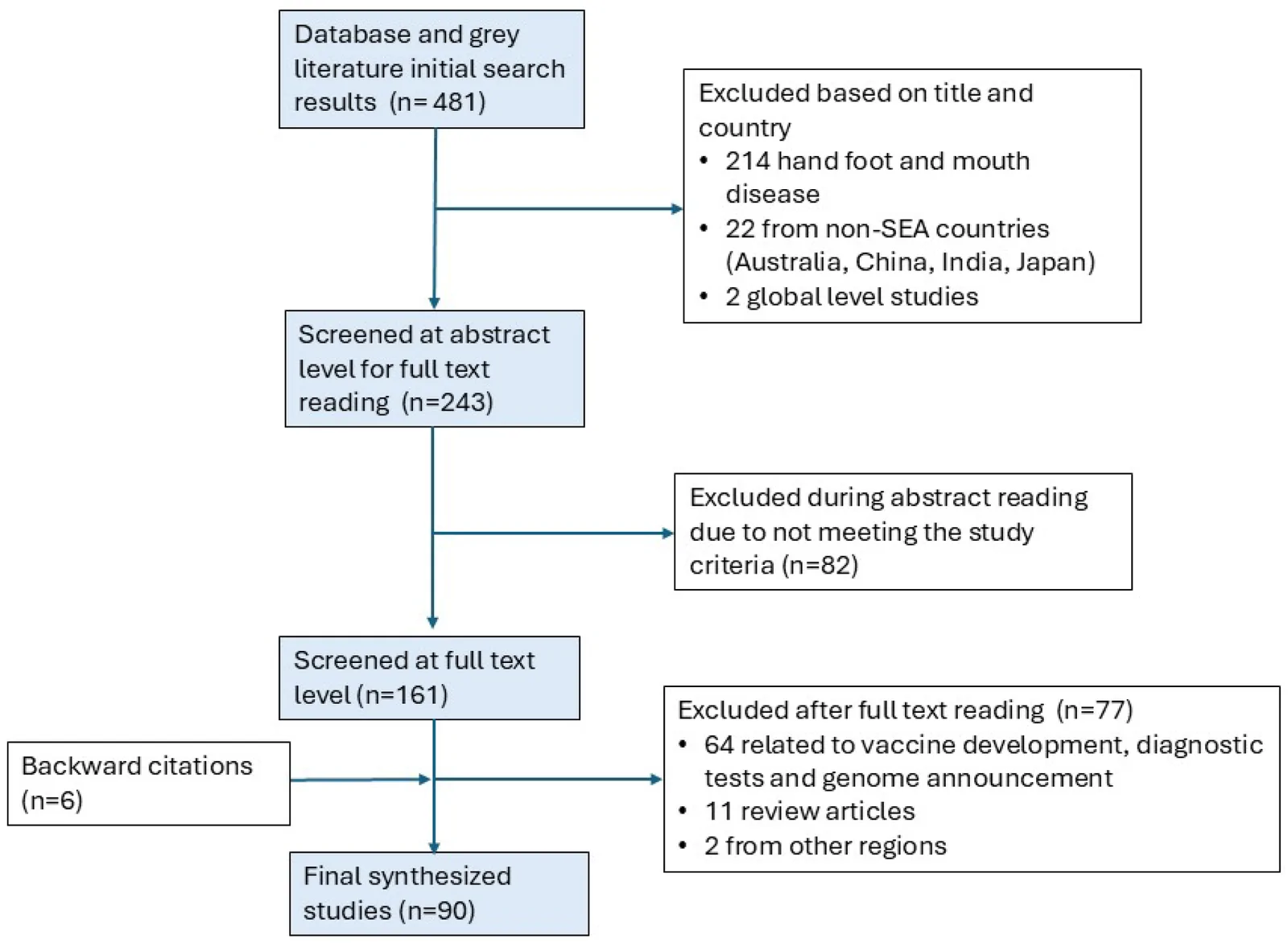
Flow diagram of the literature search for title screening, abstract selection, and the final full-text reading.

Primary study screening and eligibility assessment were performed by the first author. Following the initial search that generated 481 publications (Figure 1), 243 studies were selected after removing 214 articles with titles including ‘hand, foot and mouth disease’, 22 articles from countries that are not within SEA, and two global-level studies. Review of study abstracts eliminated 82 articles that did not meet the study criteria, decreasing the pool to 161 articles. Review of the full texts of articles led to the elimination of eleven review articles; 64 articles related to vaccine development, diagnostic tests, and genome announcements; and two additional studies from other regions. To supplement the search, the reference lists of included studies were screened using backward citation searching to identify additional relevant literature. We also did not consider published comments or articles written in languages other than English. This further reduced the number of included articles to a final quantity of 90 articles. To improve consistency, the reviewer applied predefined eligibility criteria throughout the screening process and reassessed studies for which eligibility was uncertain before making a final selection.

The findings related to each country, title, author, required evidence, epidemiological analytical methods, and limitations of each study are tabulated and presented for all the countries for each PCP stage and key activities (Supplementary Table S1). Two summary tables were created to present the major challenges and priorities of each PCP stage, as well as the usage, strengths and limitations of the epidemiological tools of the eight countries.

Stage 3 is pivotal for countries to move from endemic to disease-control status. As Vietnam is in Stage 3 of the PCP FMD pathway and shares land borders with Cambodia, Laos, and China, as well as maritime borders with Thailand, the Philippines, Indonesia, and Malaysia, the country is at the center of livestock movement activities. Given our longstanding collaboration with Vietnamese partners, including several joint publications [11,12,13], and access to official FMD control plans, Vietnam was selected as an illustrative case study to demonstrate the application of the proposed framework.

Based on key activities listed in the PCP guideline document titled, ‘The Progressive Control Pathway for FMD control (PCP-FMD)’ under each stage, a framework was created to present literature evidence required to fulfil each criterion. Standards of minimum and advanced evidence for each step were proposed, considering the resource setting and technical capacity. Minimum evidence was designated based on a low-resource setting indicating simpler quantitative approaches and qualitative methods. Advanced evidence was categorized based on complex quantitative analysis methods that require more training and resources, such as FMDV sequence data. The practical approach of evidence was evaluated in relation to the exemplary case study of Vietnam. Overall, the results are presented according to the analytical tools utilized, progressing from minimum to advanced.

## 3. Results

The countries within SEA are at different stages of the PCP, and scientific evidence on the achievement of various activities of each PCP stage is available [14,15,16]; however, there are other areas that still require attention, including risk-based surveillance [8]. Table 1 presents the PCP status and major challenges and priorities of the eight countries included in this review.

### 3.1. Stage 1

#### 3.1.1. Identifying FMD Risk Factors

Decades of epidemiological investigations have indicated that the occurrence and spread of contagious diseases such as FMD are not random but influenced by the presence or absence of factors related to the environment, host, and the virus identified as risk factors. Risk-factor studies provide the evidence to prioritize interventions and develop risk-based control strategies in PCP Stages 1 and 2. Descriptive statistics, case–control and cross-sectional studies utilizing multivariable logistic regression, and multivariable generalized estimating-equation models have been used in the region [22,39,46,47,48,49]. Spatial trends were accounted for in regression models in studies from Thailand [40,46,48]. Collectively, these studies identified seasonal effects; livestock populations; animal aggregating points such as livestock markets, slaughterhouses, and international borders; and farm biosecurity as major risk factors.

Due to the contagious nature of FMD, if a given administrative unit experiences a high number of outbreaks, the adjacent units may also document higher numbers of outbreaks based on their proximity to the high-risk area, making it challenging to dissect the influence of risk factors from simply proximity alone. For that reason, spatially explicit regression models are necessary to account for the spatial (and temporal) lack of independence in the observations [50]. In Vietnam, we employed a spatio-temporal Bayesian model to account for the spatio-temporal dynamics of Foot-and-Mouth Disease (FMD) outbreaks when identifying risk factors [12]. A primary limitation of these models, however, is their inability to account for long-distance, transboundary animal movements. This is a critical gap in Vietnam, where extensive livestock movement occurs from the Northern to the Southern regions [11,37]. To address this, we integrated phylogeographic analyses, which are increasingly used to reconstruct FMD virus (FMDV) dissemination and uncover movement history [51].

Alternatively, network analysis was used to determine the animal-movement risk in a risk-factor study conducted in Lao PDR, which report limitations of data for the analysis [14]. A multi-criteria decision analysis system was used to identify FMD risk factors in Thailand [52]. In this analysis, expert evaluation regarding spatial risk factors was used to determine FMD high-risk areas with spatial mapping. A sensitivity analysis identified the key risk factors influencing the model.

A limitation common to many risk-factor studies is that they rely on reported outbreak numbers, which are known to often be under-reported or reported late. A study conducted in Lao PDR accounted for this by expanding the study window beyond the reported outbreak duration within four standard incubation periods [22]. The capture–recapture method was used to determine the actual outbreak size in Thailand to account for the prevalent under-reporting of FMD outbreaks [43]. A study performed in Lao PDR emphasized the importance of reporting FMD outbreaks to the national database [53] FMD outbreaks are seasonal in endemic regions. Seasonal, climatic, and environmental factors correlate with risk factors related to husbandry systems, patterns, and timing of animal movements, as well as survivability of the virus outside the host [54,55,56]. These complex interactions should be taken into consideration rather than the individual consideration of each different variable such as in a machine learning approach [57,58].

#### 3.1.2. Virus Circulation

Seroprevalence studies are commonly used in the region to determine the FMD prevalence and risk factors as a more reliable method compared to the official outbreak reporting system and reporting only based on clinical signs [28,30,31,59]. These studies also provide evidence of subclinical viral circulation. During 2009–2013, serological monitoring of the virus across different regions in Vietnam, along with molecular epidemiological analysis of FMDV sequence data isolated from different parts of the country, supported the identification of patterns of FMDV circulation [60]. For FMD-endemic countries conducting seroprevalence studies to detect virus circulation, an important consideration is differentiation of antibodies caused by natural infection from those induced by vaccination (DIVA assays). Standard FMD vaccines are purified to remove non-structural proteins [61]. Therefore, in field conditions, measurement of antibodies against nonstructural proteins (NSPs) indicates FMD infection. It is common to have residues of nonstructural proteins when some countries locally produce live attenuated vaccines [62]. In these instances, NSP ELISA tests cannot differentiate vaccinated animals from infected animals, emphasizing the importance of quality control in FMD vaccine production [63]. The inability to differentiate vaccinated animals from naturally infected animals is a limitation in seroprevalence studies [28,30,59].

An attempt to optimize identification of FMDV circulation in Vietnam via risk-based surveillance comparing seroprevalence at slaughterhouses and farms provided differential results [11]. However, it was identified that sequences isolated from these slaughterhouses were representative of circulating strains and could be used for early detection of outbreak sequences. The analysis was performed by comparing sequences from slaughterhouses, farms, and outbreaks in a Bayesian phylogenetic analysis evolutionary tree (BEAST). Currently circulating serotype O sequences in Vietnam are related to O/ME-SA/Ind-2001, O/ME-SA/Pan Asia, and O/SEA/Mya-98 lineages, with O/ME-SA/Pan Asia being the most common. Results from this study also indicated that within these lineages, multiple distinct genetic clusters emerged, spread, and eventually disappeared over time.

Many endemic countries do not possess digital databases tracking animal movements, which limits their ability to incorporate animal movement into hypotheses about viral circulation. Few countries in SEA document livestock movements [24,64]. In the absence of animal movement data, the combination of molecular data with phylogeographic analysis enables the reconstruction of past viral movements, which are likely to be correlated with host movement. The BEAST analysis provides an approximate timeline of the emergence of different viral lineages compared to phylogenetic trees, such as maximum-likelihood or neighbor-joining trees. BEAST analysis was used to detect the first incursion of the O-Ind2001d lineage to Vietnam and O/Pan Asia circulation [37,65]. The spatial–temporal distribution of the O Cathy topotype was identified in the Philippines using BEAST analysis, as well as both serotypes (O and A) in Malaysia and in Southeast Asia in general [15,66,67,68].

Numerous studies have used neighbor-joining trees [69,70,71,72,73] and maximum-likelihood trees [35,74,75,76,77], to show the relatedness among identified sequences within Vietnam and the surrounding countries, among other findings. The neighbor-joining method is a distance-based method that combines similarity clustering to produce a single tree and is considered most appropriate for larger dataset analyses due to its low computational power. Maximum-likelihood trees are created based on the optimal branch length and the substitution parameters, with repeated selection for the best trees via bootstrap sampling, and are considered useful in smaller dataset analyses due to high computational power [78].

Recognizing circulating strains and seroprevalence is crucial for making decisions regarding vaccination and transboundary animal movement. As reflected in later PCP stages, ongoing active surveillance is necessary, as FMD is a continuously evolving RNA virus. With the high rate of emergence of recombination and frequent transboundary introductions, early detection of new strains is important to make predictions based on the evolution of the virus in FMD-endemic countries [38,79]. With the increased availability of sequencing technologies, experimental studies have explored the feasibility of PCR technology for FMDV detection in the region [80,81,82,83,84,85,86].

#### 3.1.3. Stakeholder Engagement

Stakeholder behavior strongly influences FMD surveillance and the effectiveness of control. Published studies related to stakeholder engagement from Vietnam are limited, whereas other countries in SEA have reported multiple studies based on numerous analytical methods. Stakeholder engagement analyses are primarily used to evaluate existing FMDV surveillance systems and farmers’ tendency to report FMD outbreaks and to determine disease control measures such as vaccination [17,18,23,43,87,88,89,90,91,92]. Common study designs include knowledge, attitudes, and practice (KAP) models and focus-group discussions. These studies have identified the lack of stakeholder willingness to report FMD outbreaks, the impact of cattle trade networks on FMD spread, and the evaluation of control strategies used in the region. Other studies in Southeast Asia have validated these methodologies. One study in Cambodia analyzed the validity of KAP methods against seroprevalence data using Bayesian methods [17]. A latent class model was used in Myanmar to compare farmer-reported outbreak histories with serological evidence of FMD exposure, thereby assessing the accuracy of verbal disease reporting [31]. The sensitivity of this analysis was affected by differences that exist in the surveillance and notification systems in different townships in Myanmar. Additional stakeholder engagement studies can help identify both legal and illegal animal movement routes and practical ways to control outbreaks, with stakeholder compliance accounting for well-known transboundary animal movement in the region.

#### 3.1.4. Socio-Economic Impact

Foot-and-mouth disease causes considerable direct and indirect economic losses at both farm and national levels [1]. These impacts are likely underestimated because of the under-reporting of outbreaks. Economic analyses conducted across SEA have highlighted the burden of FMD on both commercial and smallholder livestock producers while demonstrating vaccination programs as a major disease control strategy.

Statistical methods, such as the restricted maximum likelihood method [93] cross-correlation modeling [94], regression analysis [95,96] regression analysis with propensity score matching [97], and partial least-squares structural equation modeling [98], have been used to determine socio-economic impact using questionnaire data during FMD outbreaks. Limitations of these studies include uncertainty in indirect cost estimates, restriction to a few areas of the country, recall bias, and estimation of losses in certain types of management systems (micro vs. smallholder) only.

Cost–benefit analysis and partial budget analysis are commonly used to evaluate the economic impacts of viral diseases [99]. To determine the economic impact of FMD in Vietnam, both cost–benefit analysis at the national level and partial budget analysis at the farm level were conducted based on simulated data and data collected via questionnaires [9]. A cost–benefit analysis that considered the impact of the vaccination program was implemented in Lao PDR, and the financial impact of FMD was calculated at both national and village levels [32]. A partial budget analysis and a cost benefit analysis conducted in Cambodia to estimate the financial losses associated with FMD outbreaks and vaccination incentives among farmers [19,100]. These analyses were based on assumptions such as that FMD vaccination completely prevents FMD infection in vaccinated animals and that vaccination cost is fixed. Sensitivity analyses were carried out to determine the robustness of the analysis.

A value-chain analysis conducted in Cambodia estimated the national economic impact of FMD by adjusting reported outbreak data to estimate the likely true incidence of FMD outbreak-associated income losses. Value-chain analysis accounted for legal and illegal large ruminant trade networks in Cambodia [21]. Production losses and FMD morbidity rates were estimated in different districts in Thailand, accounting for different outbreak management systems, indicating that farm size and outbreak duration significantly affected FMD outbreak-associated income loss [101,102]. Collectively, these findings suggest that preventive vaccination and improved disease control measures can generate substantial economic benefits.

According to the PCP FMD guideline, minimum requirements of Stage 1 include description of socio-economic drivers and understanding of FMDV circulation in the country. To achieve these steps, identification of FMD risk factors using regression models and seroprevalence studies and stakeholder engagement via participatory methods may be recommended as minimum tools under low-resource settings. Ultimately, the overall aim of PCP Stage 1 is to identify the current FMD situation in the country to come up with a strategic control plan in PCP Stage 2. At present, Cambodia is in Stage 1, with the intention to reach Stage 2. Table 2 presents the minimum and advanced methods used in SEA countries to show the achievements of each stage.

### 3.2. Stage 2

The aim of Stage 2 is to identify a zone/husbandry system in the country that could reduce the impact of FMD. Epidemiological identification of risk hotspots where outbreaks originate/emerge contributes towards this goal.

#### 3.2.1. Identifying Important Risk Hotspots for FMD Transmission to Implement FMD Control Measures

According to the PCP pathway, by Stage 2, a strategic control plan should be in place for countries aiming at FMD freedom. The identified high-risk areas, risk factors, and circulation of the virus support the improvement of strategic control plans. High-FMD-risk areas are defined by relatively high case numbers (e.g., high prevalence and incidence). Identifying high-risk areas is necessary for the deployment of control measures such as targeted vaccination and surveillance prioritization in resource-limited settings.

Spatial–temporal cluster analysis detects areas where more or fewer outbreaks are recorded relative to null expectations. Several studies have identified spatial–temporal clusters of high-risk FMD areas in the region via SaTScan analysis, including Thailand [54], Vietnam [55], Indonesia [34] and the whole region [103]. Except for a few island nations, geographical land borders connect most countries in Southeast Asia. In Lao PDR, high-risk areas were identified by standardized morbidity-rate calculation and a Besag spatial model accounting for the spatial autocorrelation [104].

As discussed earlier, in Vietnam, incorporating phylogeographic inference into a spatial–temporal Bayesian model, high-risk areas were identified in the northern and southern parts of the country, where transboundary animal movement is common, particularly from Cambodia and Malaysia to Vietnam [12]. Viral movement inferred by such analyses reflects underlying patterns of host movement that ultimately drive patterns of population connectivity and therefore FMD risk [51]. The reported case count was used as the outcome of this model, as opposed to the reported number of outbreaks, which could allow a single large outbreak to have too much influence on the results, whereas outbreak counts treat large and small outbreaks equally.

In Thailand, a multi-criteria decision analysis and machine learning methods were used to predict FMD high-risk areas [52,57]. In the machine learning analysis, only the northern region of Thailand was represented, which may limit the generalizability of the results.

#### 3.2.2. FMDV Transmission Among Species

In PCP Stage 2 activities, countries are expected to plan for the elimination of FMD from at least one husbandry system. Identifying interspecies FMD transmission is important to achieve this goal. A previous study performed in Vietnam identified FMDV transmission from cattle to pigs and pigs to cattle [37]. A different global-level study showed transmission from cattle to pigs and buffalo [105]. Both studies used discrete phylogeographic trait analysis. Performing the same analysis with a larger number of sequence data (240 sequences—160 from cattle and buffaloes and 80 from pigs), we identified that the virus was mainly transmitted from cattle to pigs (Bayes factor = 77, 512.2), with less evidence of transmission from pigs to cattle (Bayes factor = 0.51), indicating strong support for transmission from cattle to pigs [12]. In Vietnam, FMD surveillance in small ruminants has not been carried out (Vietnam FMD National Control Program, 2025). In general, limited sequence data hinder the identification of FMD transmission dynamics and therefore species-specific control strategies. Inconclusive interspecies transmission evidence emphasizes the need for further studies with more sequence data to support this activity. Although small ruminants play a major role in meat production in the region, few FMDV sequences were available from small ruminants.

A study using a spatial transmission kernel considering daily outbreak data from Vietnam identified cattle and buffalo as having a larger impact on disease spread than pigs [36]. This could be because pigs are reared for a shorter duration compared to cattle and buffalo in the country [106].

#### 3.2.3. Impact of Biosecurity

Although biosecurity is an essential component of FMD prevention and control, it has received relatively limited research attention in Southeast Asia. A case study published in 2019 proposed a set of biosecurity measures for vaccination campaigns in the region based on a literature review, stakeholder workshops, and field exercises [92]. In Cambodia, a knowledge, attitudes, and practices (KAP) study assessed biosecurity practices among smallholder livestock farmers and identified low baseline awareness and poor adherence to basic biosecurity measures [107]. The findings suggested that deficiencies in biosecurity knowledge and implementation may contribute to continued FMD transmission in smallholder production systems in the region.

#### 3.2.4. Impacts of FMD Vaccination Programs and Other Control Measures

Across SEA, the evaluation of FMD control programs spans diverse methods, ranging from socio-economic assessments to epidemiological modeling. Considering socio-economic aspects, research has focused on stakeholder involvement. Participatory methods and mean importance scores have been utilized to evaluate farmers’ satisfaction with government vaccination programs in Vietnam and Indonesia [108,109]. The value of these findings is constrained by a reliance on purposive sampling, which limits broader generalizability.

Beyond stakeholder perceptions, monitoring the impact of vaccination requires quantitative methods. Regression analyses have been deployed to measure vaccine impact in Lao PDR, demonstrating the impact of risk-based partial vaccination strategies and regular vaccination [25,26,27]. A predictive simulation model used in Thailand evaluated the impact of varied FMD control strategies such as culling, ring vaccination, and animal movement restrictions [110].

Assessment of diagnostic accuracy is vital for post-vaccination monitoring. To this end, Bayesian latent-class analysis has evaluated test sensitivities and specificities of the non-structural protein (NSP) ELISA, virus neutralization test (VNT), a solid-phase competitive ELISA (SPCE), and a liquid-phase blocking ELISA (LPBE) tests, accounting for missing data in Lao PDR [29] and regionally for 3ABC diagnostic tests across multiple SEA nations [111]. Regional studies have also highlighted the importance of logistics associated with vaccination programs. In Cambodia, a factorial design was utilized to demonstrate how vaccine storage conditions directly influence serological response, highlighting the critical role of cold-chain maintenance [112].

Vietnam enforces vaccination in control zones within border provinces, adjacent buffer zones, and low-risk zones (FMD national control program, Vietnam). In control and buffer zones, where 80% vaccination coverage is implemented, mainly cattle, buffalo, and pigs are targeted [9,108]. Since 2018, Vietnam has started to produce monovalent type-O FMD vaccines using local strains. According to the 2021–2022 WOAH evaluation report, Vietnam has been conducting post-vaccination monitoring by estimating vaccination coverage and serological monitoring (Vietnam FMD National Control Program, 2025). Except for government sources, no published studies are available regarding post-vaccination monitoring in Vietnam or other countries. At Stage 3 of the PCP, it is important to quantitatively determine adequate vaccine effectiveness and coverage at the village or commune level to maintain FMD-free zones. Published studies related to the evaluation of risk-based control measures in targeted zones are not available from the region.

Assessment of the FMD control program at Stage 2 is crucial to progress toward Stage 3 and beyond. When it is not economically feasible to implement countrywide control measures, it is important to focus on high-risk areas. Minimum requirements of Stage 2 include the implementation of risk-based control measures in target zones that can be identified via SaTScan analysis and evaluation of the impact of vaccination program evaluated by multi-variable logistic regression models as minimum tools under low-resource settings.

### 3.3. Stage 3

Stages 3 and 4 mainly focus on progression towards the prevention of endemic FMDV circulation. This could be demonstrated through reductions in FMD incidence in domestic animals in one zone of the country and ongoing monitoring of circulating strains. At this stage, earlier implemented control measures are refined to be effective, including striving for early detection of outbreaks.

#### 3.3.1. Ongoing Monitoring of Circulating Strains and Risk in Different Husbandry Systems

Progressing through Stages 1–3 of the PCP requires the identification of circulating FMD strains using samples representative of different production systems and geographic regions. Enhancing surveillance capacity at Stage 3 remains fundamental to controlling transboundary animal diseases like FMD [113]. While surveillance is defined as the systematic and ongoing observation of disease distribution [114], passive surveillance remains the status quo in many endemic countries. Under this system, clinical outbreaks are reported locally before escalating to district and central veterinary offices, where confirmatory diagnosis may or may not be carried out later. Recent evaluations in Southeast Asia incorporating lineage reports, surveys, and stochastic simulations indicated that passive systems have low sensitivity and may fail to detect less prevalent serotypes, such as Asia 1 [115].

Population-level virus circulation is best determined via active farm surveillance, which is a requirement of this stage. While maintaining the activities in Stages 1 and 2, an additional goal of Stage 3 is the rapid detection of outbreaks in at least one zone of the country. Active molecular surveillance for FMDV is widely documented in Vietnam [37,65]. Routine active surveillance at farms that represent the population level may not be cost-effective due to increased biosecurity hazards, ethical aspects, and increased labor investment. For FMD, clinical signs are not pathognomonic, and farmers may be unaware during the early period of spread in their farms; thus, early detection of diseases is challenging in farm settings [31,116]. However, conducting surveillance at points where animals from several farms congregate (such as markets or slaughterhouses) may provide robust and cost-efficient sentinel surveillance that could both identify circulating strains and provide sequence data for molecular epidemiological studies that would support other aspects of the PCP [53,117].

The genetic diversity of FMDVs isolated from apparently healthy animals at slaughterhouses is representative of the diversity of FMDVs isolated from the source population (i.e., farms) [11,118]. Apart from Vietnam, slaughterhouse surveillance has been implemented in other countries in the region, such as Cambodia [119], Laos and Myanmar [53,117]. This is an important step for rapid outbreak detection in FMD-free zones when a country is in Stage 3.

In FMD-endemic countries, FMD carrier cattle and buffalo are common, which complicates the achievement of FMD-free status. Once infected, these animals harbor the virus without exhibiting clinical signs. Both vaccinated and non-vaccinated cattle and buffalo become carriers. Carriers can only be identified by probang samples [120] and are overlooked in routine surveillance and active surveillance with serology data. Carrier animals are known to harbor recombinant viruses that support FMDV evolution [121] and are speculated to maintain the virus for extended periods to initiate new outbreaks [122]. It is uncertain if carrier animals can infect other animals, but the focus is more on the persistence of FMDV in the population [120,123]. Most routine surveillance systems fail to identify carrier animals. Therefore, the incorporation of targeted probang sampling in high-risk zones post outbreak investigation for carrier detection is an essential step towards the achievement of complete FMD freedom in Stages 4 and 5.

#### 3.3.2. The Incidence of Clinical FMD Is Progressively Eliminated in Domestic Animals or at Least One Zone in the Country

To reduce clinical FMD in at least one zone in the country by improving herd immunity, it is important to determine the reproduction number (R0) of FMD during an outbreak. The average number of secondary infections caused by one typical infectious individual in a fully susceptible fixed population during its entire infectious period is known as R0 [124]. A given population is not fully susceptible to an infectious disease due to maternal immunity, immunity from previous infections, and vaccination. The effective reproductive number (Re) is calculated by accounting for such variables [125,126]. Re values closer to one indicate endemic status, and anything above one will be reported during ongoing outbreaks. Whilst the Re value is calculated with continuing outbreaks (time-variant R value), it would be most helpful in outbreak control, as it provides an opportunity to evaluate and modify control measures. However, the R value needs to be considered in combination with several additional variables to be useful in FMD control.

R0 has been calculated in different identified spatial–temporal clusters in Vietnam using five different methods [13]. For all clusters, the median R-value remained between 1.25 and 1.61. An R-value closer to 1 indicates the endemic status of FMD in Vietnam. At the individual cluster level, it is evident that the R-value is higher in north Vietnam than the south. Similar findings were observed in a different study conducted in Vietnam using a spatial transmission kernel model with reported outbreak data from 2007 [36]. These clusters are unlikely to change with time due to associated economic factors and animal movement patterns occurring through Vietnam to China, from Malaysia, Cambodia, and Laos [33]. The effective reproductive number at the subdistrict level has been calculated in northern Thailand using the epidemic doubling-time method [127], a transmission kernel [128], and the trade network at the Thailand-Myanmar border [129]. Under-reporting and timely reporting of outbreaks impact calculations of the effective reproductive number.

Forecasting approaches support FMD incidence reduction by enabling timely implementation of control measures. In Thailand, several time-series methods, including the Seasonal Autoregressive Integrated Moving Average (SARIMA), Error Trend Seasonality (ETS), and Neural Network Autoregression (NNAR), were applied to characterize temporal trends and seasonal patterns of FMD outbreaks between 2010 and 2020 [56]. Despite potential under-reporting of outbreaks, the study identified a seasonal pattern, with FMD incidence peaking between September and November, as well as an overall increasing trend in outbreak occurrence over time. These findings demonstrate the value of forecasting tools in informing risk-based surveillance and the timely allocation of disease control resources such as vaccination.

Stage 3 completion requires outstanding active and passive FMD surveillance and the rapid identification of new viral incursions. To meet these standards, evidence of active surveillance integrating serology and phylogenetic data analysis, as well as R0 calculation, to determine the intensity of outbreaks in high-risk areas stands as a minimum tool under low-resource settings.

### 3.4. Stage 4

#### Identifying the Risk Pathways for New Viral Incursion

Once countries have completed the requirements of Stages 1 to 3, they may request official endorsement of their FMD control program from the WOAH to proceed to Stage 4. The intention at Stage 4 is to eradicate FMD from a zone, a husbandry system, or the entire domestic livestock population through high-quality surveillance. At this stage, assessing the risk of FMD incursion into FMD-free areas from other countries and wildlife to maintain FMD-free status with vaccination is essential. Relying on the baseline requirements of previous stages does not guarantee advancement to Stage 4, as evidenced by Vietnam’s current effort to achieve this goal. Due to the inherent complexities associated with FMD, such as the circulation of multiple serotypes that do not offer cross-protection [130], viral persistence in animals without clinical signs as carriers [120], FMD vaccination not offering long-lasting immunity [131], and the lack of a regional approach to control FMD, it is challenging for countries to reach and maintain later PCP stages. Recent studies addressing the wildlife–livestock FMD interface were not available from SEA.

Risk assessment studies have been conducted in the region to assess the impact of transboundary animal movement from Myanmar to Thailand, Laos to China, and to the Philippines via imported dairy cattle from Brazil [40,41,45]. According to the SEACFMD 27th meeting updates, Vietnam, Indonesia, Malaysia, Lao PDR, Myanmar, and Thailand have proposed FMD-free zones. The monitoring of both transboundary and domestic movements of animals is a critical step for disease control and preparedness. Transboundary and trans-pool spread of FMDV has been extensively documented in the region [38,105].

Distinct areas across 34 provinces in Vietnam have been proposed to be FMD-free zones (Vietnam FMD National Control Program, 2025). In Vietnam, it is operationally difficult to maintain FMD-free zones due to animal movement and inadequate surveillance and vaccination. So far within SEA, only Thailand has reached Stage 4 of the PCP. The eastern region of Thailand has been proposed as an FMD-free zone. The lack of a countrywide FMD surveillance system and inadequate stakeholder engagement were identified as limitations with respect to FMD-free zones in the country [40]. Therefore, evaluating stakeholder engagement from Stage 1 onward is essential for the strengthening of surveillance systems and the improvement of compliance with animal movement controls. A framework to evaluate FMD control policies in FMD-free zones in Thailand and the Philippines has been proposed [41,44]. Overall, the evidence suggests that sustaining FMD-free zones in Southeast Asia remains a regional challenge rather than a purely national one, requiring coordinated surveillance, risk-based management of transboundary animal movement, and sustained stakeholder engagement to prevent viral reintroduction and support long-term PCP progression. Strengths and limitations of epidemiological methods described under each PCP stage are summarized in Table 3.

## 4. Discussion

This study demonstrated how epidemiological and molecular epidemiological tools assist FMD-endemic countries in SEA as they navigate through the stages of the PCP. Vietnam was used as an exemplary case study for comparison, given that is in the pivotal Stage 3, where countries achieve sustainable FMD control through national control programs. A key contribution of this review is the development of a framework linking epidemiological tools to PCP evidence requirements and resource availability. The FAO SEACFMD roadmap meeting and previous studies have identified a lack of FMD risk assessment capacity and the implementation of biosecurity measures as major concerns in the region [4,8]. Consistent with these regional priorities, we identified limited published studies on risk assessment, the evaluation of vaccination programs, and biosecurity implementation. Across SEA, transboundary livestock movement remains the primary threat to the establishment and maintenance of FMD-free zones. In Vietnam, FMDV movement from Malaysia to Vietnam and from Vietnam to China was identified [12]. Only a few countries have achieved studies on regional FMD transmission dynamics [38] and advanced analytical tools to demonstrate transboundary movement and the use of risk-based surveillance, which are crucial in later PCP stages. The strengthening of regional collaboration through existing data-sharing platforms such as the ASEAN Regional Animal Health Information System (ARAHIS) that facilitate cross-border surveillance and joint outbreak investigations is necessary to overcome these challenges.

Activities related to identifying risk factors, viral circulation, the detection of high-risk areas, and ongoing FMD surveillance described here facilitate standardized evidence for progress evaluation along the PCP that is currently inconsistent and subjective. These findings highlight the need for standardized evidence requirements while recognizing that countries may achieve PCP objectives through different combinations of epidemiological tools depending on their technical and financial capacity. Analytical tools enable policymakers to compare their country’s status with regional counterparts, assess strengths and weaknesses in current FMD control programs, and identify priority areas for improvement.

As Vietnam prepares for a potential transition to PCP Stage 4 and WOAH endorsement, further validation of national control strategies through increased surveillance and risk-based interventions is needed. To meet the requirements of Stages 1 and 2, Vietnam moved beyond descriptive analysis to predictive and economic modeling to determine stakeholder impact, high-risk areas, and viral circulation. However, limited quantitative evaluation of specific control interventions hinders us from understanding the progress made towards overall FMD transmission reduction. The national program for FMD prevention and control in Vietnam is the legal framework for FMD control in the country and is expected to be updated along with the capacity of veterinary services (Vietnam FMD National Control Program, 2025). Stage 3 activities—namely, active surveillance and identification of the efficacy of outbreak containment in target zones—have been evaluated, yet strengthening of passive surveillance remains necessary, particularly regarding timeliness and completeness. The proposed establishment of FMD-free zones across 34 provinces also presents operational challenges. We could not identify peer-reviewed studies evaluating entry risks or the national impact of interventions such as vaccination. Given the transboundary nature of FMD, effective animal movement control, diagnostic capacity, and coordination with regional control programs remain key challenges for Vietnam’s progression toward Stage 4.

In the PCP pathway, there are no specific recommendations to conduct epidemiological analyses in a predefined sequence for each stage. Countries are expected to generate evidence appropriate to their stage of progression and national context. Different epidemiological methods are acceptable for the evaluation of the progress at each stage, as the WOAH and FAO do not provide specific guidelines regarding quantitative or qualitative methods of evaluation. Smallholder production systems remain important across much of Southeast Asia, although there is considerable variation in livestock production systems. As shown in Table 1, differences in surveillance capacity, resources, and veterinary infrastructure among Southeast Asian countries that create different challenges would influence implementation of the proposed framework. Low biosecurity, unregulated animal movements, and infrastructural resource limitations are common among countries [8]. We specifically focus on key activities in Stages 1 to 3 relevant to several countries in the region. Many of these challenges, including under-reporting, limited surveillance integration, and inconsistent vaccination monitoring, are similarly reported across other endemic regions. This suggests that the proposed evidence framework may have broader applicability beyond Southeast Asia.

Advanced PCP stages require stronger analytical expertise. The SEACFMD 2021–2025 project underscored the importance of capacity building in the region. The creation of an enabling environment conducive to disease control includes the evaluation of the veterinary services of each country as part of a Performance of Veterinary Services (PVS) analysis at Stage 1 of the PCP. Many online platforms are available to support capacity building in the region, such as the Asia Pacific Consortium of Veterinary Epidemiology [134], the Food and Agriculture Organization [135], the World Organization for Animal Health [136], and Progress Vet [137]. These platforms support the expansion of knowledge of veterinary epidemiology and disease surveillance among veterinarians in the region to meet the requirements of later PCP stages. Several of these platforms provide step-by-step chapters to expand veterinarians’ knowledge from fundamental competency related to disease control (such as measures of disease frequency, diagnostic testing, and space–time interaction of disease occurrence), outbreak investigation, and surveillance data analysis to risk assessment with self-evaluation exercises. Training and educating veterinarians may help ensure compliance among other stakeholders in disease control activities (e.g., enhancing biosecurity on farms [107], surveillance [113], national policy updates, and fulfilling the criteria of the Performance of Veterinary Services (PVS) conducted by the WOAH).

To the best of our knowledge, this is the first review to systematically link epidemiological tools to evidence requirements across different stages of the Progressive Control Pathway (PCP) by synthesizing information from both peer-reviewed and gray literature sources. However, several limitations should be acknowledged, including restricted access to unpublished governmental data, the exclusion of literature published in languages other than English, and inconsistencies in PCP stage reporting. As this was a narrative review, the literature search was not intended to be exhaustive. The literature search was conducted using only the Web of Science database, which may have resulted in the omission of relevant studies indexed in other databases or published in regional journals during study screening. Selection was conducted by a single reviewer, which may have introduced selection bias and increased the possibility of inadvertently excluding relevant studies. These limitations may have reduced the comprehensiveness of the identified evidence. However, the search strategy was developed systematically and supplemented by reference-list screening and review of relevant government and organizational documents to improve the identification of relevant evidence.

This study supports the translation of the theoretical PCP framework into practical, evidence-based guidance for endemic settings based on available evidence from SEA. We specifically focused on key activities in Stages 1 to 3 relevant to several countries in the region. There is a common lack of evidence related to biosecurity, enhanced surveillance, and risk analysis. Progression through the PCP will require not only national investments in these domains and analytical capacity but also stronger regional collaboration to support data-sharing platforms, harmonized surveillance approaches, outbreak investigation and the generation of minimum evidence required for PCP advancement. By supporting efforts to bridge the gap between research and policy, this review provides policymakers with a practical framework to support evidence-based decision-making and accelerate progress toward sustainable FMD control and eventual FMD-free status.

## Figures and Tables

**Table 1:** Challenges and priorities faced by Southeast Asian countries across different Progressive Control Pathway (PCP) stages.

Country	PCP Stage	Primary Strategic Priorities	Principal Implementation Challenges
Cambodia	Stage 1	Identify risk factors and high-risk areas to compile a national risk-based strategic control plan [17,18,19].	Improve the national passive surveillance system [17]. Maintain vaccination program records [18]. Under-reporting of outbreaks [19,20]. Low baseline farm-level biosecurity awareness among smallholder farmers [21].
Lao PDR	Stage 2	Improve smallholder farm biosecurity and outbreak reporting [22,23]. Establish a livestock movement database [24]. Deploy targeted, risk-based partial vaccination campaigns [25,26,27].	Under-reporting of outbreaks [22,23]. Post-vaccination monitoring [28,29].
Myanmar	Stage 2	Identify high-risk townships and pathways for risk-based vaccination [30,31]. Quantify national and village-level economic burdens to justify public investment in FMD control [32].	Inconsistencies in outbreak reporting across different townships [31]. Controlling unregulated transboundary livestock trade flows across international land borders [33].
Indonesia	Stage 2	Mitigate and suppress the major FMD re-emergence that began in 2022 after 32 years of disease-free status [34,35]. Optimize targeted vaccination programs and stakeholder feedback on FMD control.	Containing localized spatial–temporal outbreak clusters on Java Island [34].
Vietnam	Stage 3	Validate national control strategies and establish proposed FMD-free zones [36]. [Vietnam FMD National Control Program, 2025] Post-vaccination monitoring to demonstrate herd immunity.	Managing long-distance domestic livestock trade routes (Northern to Southern provinces) [11,37]. Controlling transboundary movement across international land borders [12,38].
Malaysia	Stage 3	Strengthen stakeholder engagement and farmer reporting compliance [39]. Economic impact evaluation.	Manage the ongoing co-circulation of distinct serotype O and serotype A lineages [15]. Data limitations for epidemiological studies [39].
Thailand	Stage 4	Establish and defend proposed FMD-free zones with vaccination [40,41]. Implement border biosecurity and quarantine checkpoints [42].	Mitigating transboundary incursion risks, especially along the Thailand–Myanmar border [42]. Outbreak under-reporting [43]. Inadequate stakeholder engagement [40].
The Philippines	FMD-Free	Maintain an official WOAH-endorsed FMD-free status without vaccination [44,45]. Quantitatively model and mitigate potential reintroduction pathways.	Maintaining strict vigilance and serological monitoring in historical surveillance buffer zones [44]. Risks of FMD entry via live cattle imports. [45].

**Table 2:** Proposed minimum and advanced evidence for each stage based on studies conducted in SEA countries.

Stage	Key Outcome	Advanced Evidence	Minimum Evidence
1	The distribution of FMD in the country is well described, and a ‘working hypothesis’ of how FMD virus circulates in the country has been developed.	Models accounting for spatial temporal autocorrelation Machine learning models	Logistic regression models
	Identification of the most common circulating strains of FMDV.	Phylogenetic data analysis	Seroprevalence studies
	Estimation of socio-economic impacts of FMD on different stakeholders.	Partial budget and cost benefit analysis	Logistic regression models
	The livestock marketing network and associated socio-economic drivers are well described for FMD-susceptible species (value-chain analysis).	KAP and participatory methods, value chain analysis	Questionnaire-based descriptive analysis to identify stakeholder perspectives
2	Ongoing monitoring of circulating strains and risk in different husbandry systems.	Phylogeographic trait analysis, Spatial transmission kernel	
	Identify FMD risk zones for targeted control measures.	Spatial temporal models	SaTScan cluster analysis
	Measure the impact of control measures by reduced FMD incidence.	Predictive simulation model, regression analysis	Questionnaire-based descriptive analysis
	Implementation and impact of biosecurity measures.	KAP study	Questionnaire-based descriptive analysis
3	Ongoing monitoring of circulating strains and risk in different husbandry systems.	Sentinel surveillance phylogenetic data analysis	Serological data analysis
	Rapid detection and response for FMD outbreaks in at least one zone of the country.	Time series analysis, network analysis	Reproduction-number calculation
	The incidence of clinical FMD is progressively eliminated in domestic animals in at least one zone in the country.	Time series, spatial temporal analysis	NA
4	The risk of FMD entering the country or zone is mitigated.	FMDV entry risk assessment Phylogeographic trait analysis	NA
	FMD-free with vaccination status.	Opportunistic and structured sero surveillance analysis	NA

**Table 3:** Strengths and limitations of epidemiological tools applied at different steps of each Progressive Control Pathway (PCP) stage.

Stage	Approach	Key Application	Strengths	Limitations	Reference
Stage 1	Multivariable logistic regression	In case–control or cross-sectional studies to identify risk factors; evaluate economic impact and control measures	Adjusts for multiple confounders simultaneously	Observational association does not equal causation	[46,47,48,49,95,96,132]
Stage 1	Multivariable generalized estimating-equation model	Analyze risk factors	Accounts for data clustering	Recall and selection biases, subject to multicollinearity	[22,25]
Stage 1	Crude odds ratio	Analyze risk factors	Low resource requirements	Overestimation of risk and confounding	[39,133]
Stage 1	Seroprevalence	Estimate FMD prevalence and risk factors	More reliable compared to outbreak reports	Limited sample size	[28,30,31,59]
Stage 1	Network analysis	Identify villages with high livestock movement	Quantifies animal movement	Identified high centrality nodes depend on data availability	[14]
Stage 1	KAP & Participatory Surveys	Assesses stakeholder perspectives.	Directly captures smallholder perspectives	Recall and selection biases; limited number of variables tested	[18,97,107]
Stage 1	Phylogenetic & BEAST Analysis	Reconstruct viral lineage evolutionary histories	Acts as a genetic proxy for animal movements in the absence of digital livestock trade databases	Subject to convenient sampling biases; limited sample size.	[15,66,67,68,69,70,71,72,73,74,75,76,77]
Stage 1	Questionnaire-Based Descriptive Analysis	Risk factors, stakeholder perspective surveys and economic analysis	Simple and rapid implementation	Under-reporting of outbreaks and subjective farmer estimates	[17,18,23,43,87,88,89,90,91,92]
Stage 1	Value-Chain Analysis	Map marketing networks and drivers of animal movement	Accounts for legal and illegal trade networks	Difficulty in tracking illegal or unmonitored cross-border trade accurately	[21]
Stage 2	SaTScan Spatio-Temporal Cluster Analysis	Detect geographic areas and time windows where more outbreaks occur relative to null expectations	Directly identifies priority zones for targeted vaccination	Restricted by strict predefined geometric boundaries (circles)	[34,54,55,103]
Stage 2	Spatially Explicit Besag Models	Map high-risk zones while controlling for spatial correlation	Optimizes vaccination and surveillance at statistically verified hotspots	Results can be skewed by localized outbreaks in adjacent areas	[104]
Stage 2	Spatial–temporal Bayesian models	Map high-risk zones	Accounts for both spatial and temporal autocorrelation	Inability to account for long-distance, transboundary animal movements	[12]
Stage 2	Predictive Machine Learning Models	Synthesize environmental and husbandry interactions to predict outbreak occurrences and determine high-risk areas	Handles non-linear relationships across datasets	Lacks generalizability when applied outside the specific training region	[57]
Stage 2	Multi-Criteria Decision Analysis (MCDA)	Expert evaluations of key risk factors to generate localized risk maps	Useful in data-scarce settings	Only a selected subset of risk factors can be evaluated	[52,89]
Stage 3	Predictive Simulation Models	Quantitaively assess the impact of control measures	Provides baseline information related to implementing control measures	Assumptions related to homogenous baseline immunity	[102]
Stage 3	Active Sentinel Slaughterhouse Surveillance	Sample apparently healthy animals at major livestock congregation points to capture circulating viral diversity	Avoids ethical constraints of routine farm sampling	Subject to convenience sampling	[11,118,119]
Stage 3	Outbreak Reproduction Number (R0/Re)	Calculate transmission intensity across spatial–temporal clusters	Time-variant Re provides real-time evaluations to validate ongoing outbreak control measures	Sensitive to reporting delays	[13,127]
Stage 3	Time-Series Forecasting	Model temporal trends and seasonal outbreak peaks to predict future outbreak episodes	Enables timely allocation of disease control resources	Requires long, consistent chronological data series	[56]
Stage 4	Stochastic Trade Network Modeling	Simulate live animal flows and model risk-mitigation strategies	Allows for quantitative testing of control activities before physical enforcement	Highly uncertain due to unmonitored or illegal transboundary movements	[42]
Stage 4	Quantitative Incursion Risk Assessments	Model the likelihood of FMD introduction via live animal imports or transboundary trade pathways	Provides transparent evidence required for international WOAH endorsement of disease-free status	Constrained by severe lack of data on transboundary volumes	[40,41,45]

## Data Availability

No new data were created or analyzed in this study. Data sharing is not applicable to this article.

## Supplementary Materials

The following supporting information can be downloaded at: https://www.mdpi.com/article/10.3390/pathogens15090893/s1, Table S1: Summary of Journal Articles by PCP Stage and Key Activities, Including Evidence Requirements, Epidemiological Analytical Methods, and Limitations.
